# Preventative interventions that target cardiovascular dysfunction in children and young people: a systematic review of their effectiveness and an investigation of sexual dimorphism

**DOI:** 10.1038/s41371-022-00780-z

**Published:** 2022-12-03

**Authors:** Sophie Edwards, Murray Foster, S. Faisal Ahmed, Angela K. Lucas-Herald

**Affiliations:** Developmental Endocrinology Research Group, University of Glasgow, Royal Hospital for Children, Glasgow, UK

**Keywords:** Disease prevention, Cardiovascular diseases

## Abstract

Given that cardiovascular diseases remain a primary cause of mortality and morbidity, there is a need to consider preventative strategies to improve vascular function from early in life. The aims of this study were therefore to investigate which interventions may improve endothelial function, intima media thickness and arterial stiffness in children and young people and to assess whether these interventions differ in boys and girls. A systematic literature search of Science Direct, Pubmed, Google Scholar and the Cochrane Library by two independent reviewers was performed to source articles. Inclusion criteria were any studies including any child ≤18 years of age receiving an intervention, which measured vascular function other than blood pressure. Exclusion criteria were studies assessing children with chronic medical conditions. A total of 72 studies were identified, which met the inclusion criteria. A measurable change in outcome was more likely to be reported in studies investigating endothelial function (*p* = 0.03). Interventions which improved vascular function included physical activity and dietary programmes. Under 10% of studies considered sex differences. In conclusion, school-based physical activity interventions are most likely to result in improvements in vascular function. Endothelial function may be the first variable of vascular function to change secondary to an intervention. Standardisation of reporting of differences between the sexes is essential to be able to ensure interventions are equally effective for boys and girls.

## Introduction

Cardiovascular diseases remain the primary cause of mortality and a major cause of morbidity globally [1]. Risk factors such as obesity, hypertension and atherogenic lipid profiles are rising in children, with this rise being attributed to pregnancy complications, genetic inheritance and lifestyle and environment in childhood and adolescence [2]. Sexual dimorphism is seen in many important physiological mechanisms for the development of cardiovascular disease, although the underlying reasons behind why females and males should have these differences have not yet been fully elucidated. Mechanisms may include developmental programming, differences in sex steroid production, oxidative stress and calcium signalling [3]. There are now standardised ways to assess vascular function in children, resulting in international collaborations to establish reference ranges, which are age- and sex-matched and validated in large population datasets [4] and mitigation of cardiovascular risk in childhood has been shown to improve outcomes later in life [5–7]. As such, there is a need to assess which interventions are most effective in childhood and adolescence to improve vascular health, prior to their implementation through large-scale public health campaigns.

The primary aim of this study was, therefore, to investigate which interventions may improve endothelial function, intima media thickness (IMT) and arterial stiffness in healthy children and young people under the age of 18 years. The secondary aim was to assess whether these interventions differ in boys and girls. Endothelial function, IMT and arterial stiffness were chosen as they represent the most standardised and commonly used methods for assessing vascular structure and function in children and young people to date.

## Methods

A systematic review was conducted following the Preferred Reporting Items for Systematic Reviews and Meta-Analyses (PRISMA) reporting guidance. Studies had to fulfil the following criteria as per the PICO principle to be eligible. These were determined at the start of the project:

- P: Participant: Children and young people ≤18 years of age at time of recruitment
- I: Intervention: Any
- C: Comparison of effect to improve vascular function
- O: Outcome: Improvement in arterial stiffness, endothelial function and IMT

A systematic computerised literature search of Science Direct (previously Embase), PubMed, Google Scholar and the Cochrane Library by two independent reviewers (SE, MF) was performed to source articles in October 2021. The following key terms were searched for: (Vascular OR carotid intima media thickness OR Atherosclerosis OR pulse wave OR flow mediated dilatation OR FMD OR endothelial OR arterial stiffness OR remodelling) AND (Child OR youth). Bibliographic references were manually searched for potentially relevant studies based on the inclusion and exclusion criteria.

### Study inclusion

Two authors (SE, MF) independently reviewed the titles, abstracts and bodies of all studies identified by the search in a sequential fashion, to identify which were eligible for inclusion using the Covidence platform (www.covidence.org). After independent evaluation at each stage, the authors convened and discussed which articles should be included. Disagreements were evaluated by a third author (ALH).

The authors initially screened titles, abstracts and then full text to inform decisions on inclusion. For inclusion in the review, studies had to include participants who were aged ≤18 years of age. Studies were included that used an intervention of any kind with the expectation of improving vascular outcome, and which assessed an effect of any measure of vascular function. Only original research was included. Studies which assessed the effects of interventions within any chronic health condition e.g., type 1 diabetes were excluded. There were no language restrictions imposed.

### Data extracted

Two authors (SE, MF) independently extracted the data from the studies that met the inclusion criteria and quality standards using the Covidence review software. Data extracted included; authors, year and location of studies, type of study, setting, number of participants (sex, age), type of intervention, follow up period and measures of vascular function.

### Quality review

Assessment of bias was undertaken using the Cochrane tool, available on the Covidence platform, which assesses the methodological quality of the papers, as well as incomplete data, outcome reporting and other sources of bias. This was again performed independently by two reviewers (SE, MF). Disagreements were resolved by a third reviewer (ALH).

## Results

### Study selection

**Fig. 1:**
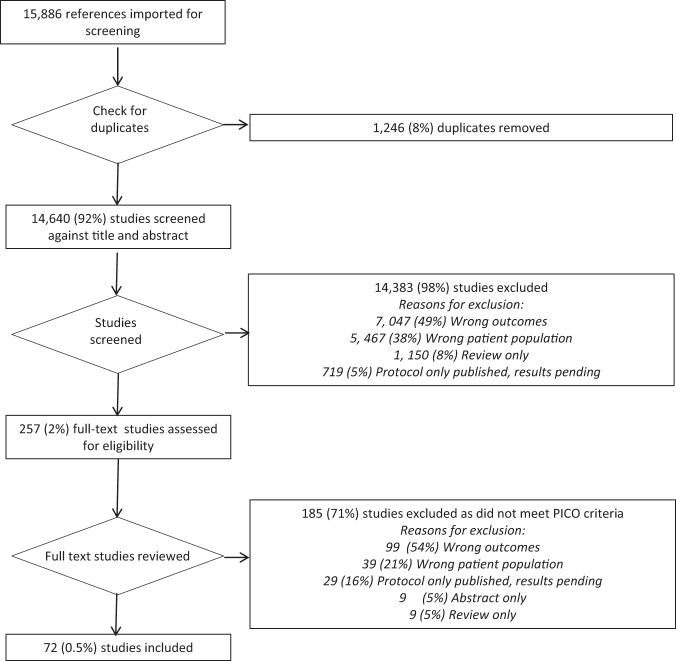
Consort diagram of included and excluded articles.

Figure 1 demonstrates the PRISMA flow diagram with the numbers of included and excluded studies at each step of the review. A high number of studies was initially identified (14,640) using the search strategy above after removal of duplicates. A total of 98% of these however did not meet the PICO eligibility criteria and as such were excluded. Thereafter, 257 full text articles were reviewed. Of the 184 (70%) excluded at this stage, 99 (54%) were due to irrelevant outcomes being reported; 38 (21%) were due to the wrong patient population (adult/chronic health condition) being included; 29 (16%) were because the protocol only had been published, with the results pending; 9 (5%) were because only conference abstracts with insufficient data were available and 9 (5%) were because they were review articles only and did not contain original data.

### Characteristics of included studies

Of the 72 studies included (Table 1), the median (range) number of participants was 60 (11, 1826) and the median age of participants was 12.1 years (0.8, 21.6). The dates of publication ranged from 2004–2021 and they were published from 29 countries in 5 continents with a total of 15,757 children studied. In terms of study design, 39 (53%) were randomised controlled trials, whereas 26 (37%) were prospective cohort studies, 5 (7%) were non-randomised controlled studies and 3 (4%) reviewed retrospective data. The majority of the studies were assessed as having a ‘medium’ risk of bias (*n* = 48, 65%).

**Table 1:** Description of included studies.

1st author	Date	Country of origin	Type of study	Number (% ⧬)	Mean age (yrs)	Intervention	Length of intervention (weeks)	Vascular outcome measures	Bias
Gruszfeld [52]	2015	Poland	RCT	1678 (16)	5	Supplement	52	CIMT	L
Mueller [30]	2017	Germany	RCT	445 (50)	11.6	Physical activity	260	RHI	M
Siegrist [48]	2018	Germany	RCT	434 (43)	11.1	Physical activity	78	Arterial elasticity	L
Arnberg [50]	2013	Denmark	RCT	203 (62)	13.5	Nutrition	12	PWV	M
Varshney [67]	2017	India	RCT	189 (37)	12.8	Drug	52	PWV	H
Davis [25]	2019	Georgia	RCT	175 (61)	9.7	Physical activity	32	PWV	M
Couch [56]	2021	USA	RCT	159 (56)	14	Nutrition	24	FMD	L
Hacke [46]	2019	Germany	RCT	135 (53)	4.8	Physical activity	24	PWV	M
Lee [39]	2020	USA	RCT	118 (64)	14	Physical activity	24	CIMT, PWV	L
Ketelhut [43]	2020	Germany	RCT	105 (51)	8.2	Physical activity	37	PWV	M
Khadilkar [75]	2012	India	RCT	90 (50)	10	Combined	16	CIMT, PWV	L
Hashemi [61]	2015	Iran	RCT	90 (61)	13.7	Supplement	4	FMD	L
Tromba [60]	2019	Italy	RCT	88 (44)	11.5	Drug	12	FMD	L
Woo [73]	2004	Hong Kong	RCT	82 (34)	10.5	Combined	6	CIMT, FMD	L
Horner [41]	2015	USA	RCT	81 (54)	14.7	Physical activity	12	CIMT, PWV	M
Ells [70]	2019	Switzerland	RCT	74 (26)	9	Combined	24	CIMT, FMD, PWV	M
Meyer [15]	2006	Germany	RCT	67 (49)	14.7	Physical activity	24	CIMT, FMD	M
Yu [65]	2013	Hong Kong	RCT	67 (100)	14	Drug	10	FMD	M
Bruyndonckx [78]	2015	Belgium	RCT	61 (75)	14	Combined	40	FMD	M
Gobbi [36]	2012	UK	RCT	59 (56)	10.6	Physical activity	10–12	CIMT	M
Ho [69]	2014	Australia	RCT	56 (55)	12.1	Drug	52	Arterial elasticity	M
Peña [63]	2007	Australia	RCT	53 (50)	13.3	Drug	8	FMD	L
Dong [66]	2010	Georgia	RCT	49 (41)	16.3	Drug	4–16	PWV	L
Chuensiri [13]	2017	Thailand	RCT	48 (0)	10	Physical activity	12	CIMT, FMD, PWV	M
Ketelhut [12]	2020	Germany	RCT	46 (45)	10.7	Physical activity	12	PWV	M
Farpour-Lambert [16]	2009	Switzerland	RCT	44 (63)	9	Physical activity	12	PWV	L
Sabri [64]	2016	Iran	RCT	40 (45)	13.1	Drug	24	CIMT, FMD	M
Karki [10]	2017	South Korea	RCT	40 (100)	15.1	Physical activity	12	PWV	M
Sung [27]	2019	South Korea	RCT	40 (100)	15	Physical activity	12	PWV	M
Yu [31]	2016	Hong Kong	RCT	38 (34)	12.3	Physical activity	10	FMD	M
Murphy [32]	2009	USA	RCT	35 (49)	10.2	Physical activity	12	FMD	M
Wong [11]	2018	USA/Korea	RCT	30 (100)	15	Physical activity	12	PWV	M
Watts [17]	2004	Australia	RCT	29 (72)	14	Physical activity	8	FMD	L
Park [14]	2012	Japan	RCT	29 (52)	12.2	Physical activity	12	CIMT	M
Ianuzzi [54]	2009	Italy	RCT	26 (39)	9	Nutrition	24	CIMT, PWV	M
Watts [18]	2005	Australia	RCT	21 (62)	9	Physical activity	8	FMD	L
Kelly [21]	2004	USA	RCT	20 (55)	10.6	Physical activity	8	FMD	M
Bassols [68]	2019	Spain	RCT	18 (7)	9.4	Drug	6–24	CIMT	L
Kelly [21]	2012	USA	RCT	11 (81)	12.7	Physical activity	24	RHI	M
van Biljon [44]	2018	South Africa	Non-randomised	109 (61)	11.5	Physical activity	6	PWV	M
Heiss [40]	2018	Germany	Non-randomised	105 (45)	12.4	Physical activity	24	CIMT, PWV	M
Donghui [77]	2019	China	Non-randomised	57 (0)	14	Combined	6	RHI	M
Poeta [22]	2013	Brazil	Non-randomised	44 (50)	10	Physical activity	12	CIMT	M
Jebeile [20]	2019	Australia	Non-randomised	30 (83)	14	Physical activity	26	CIMT, FMD, PWV	L
Kinra [53]	2020	India	Cohort	1826 (38)	21.6	Supplement	780	CIMT, PWV	M
Kinra [53]	2008	India/UK	Cohort	1165 (45)	15.8	Nutrition	780	PWV	M
Niinikoski [59]	2014	Finland	Cohort	1062 (48)	10	Nutrition	3-52	CIMT, FMD	L
Pahkala [6]	2013	Finland	Cohort	1062 (48)	10	Nutrition	–	AIMT	M
Raitakari [58]	2005	Finland	Cohort	1062 (48)	10	Nutrition	–	FMD	M
Ayer [49]	2009	Australia	Cohort	616 (49)	8	Nutrition	260	CIMT, PWV	M
Baumgartner [35]	2021	Germany	Cohort	427 (25)	14	Physical activity	104	CIMT, PWV	M
Bacauanu [33]	2019	Canada	Cohort	279 (48)	4.5	Physical activity	52	FMD	M
Böhm [38]	2012	Germany	Cohort	197 (56)	8.6	Physical activity	4	CIMT	M
Santiprabhob [76]	2018	Thailand	Cohort	115 (55)	8	Combined	52	PWV	L
Demschar [71]	2015	Germany	Cohort	98 (49)	12	Combined	6	CIMT	L
Sheridan [34]	2016	Ireland	Cohort	82 (0)	15.8	Physical activity	–	CIMT, pleth	M
Johnson [42]	2013	Canada	Cohort	82 (39)	11	Physical activity	52	CIMT, PWV	M
Böhm [37]	2012	Germany	Cohort	89 (49)	12.5	Physical activity	4	CIMT	M
Melo [45]	2016	Portugal	Cohort	79 (22)	7.4	Physical activity	–	PWV	M
Genoni [74]	2021	Italy	Cohort	55 (47)	8	Combined	52	CIMT, FMD	L
Tjønna [19]	2009	Norway	Cohort	54 (51)	14	Physical activity	52	FMD	L
Kapetanakis [51]	2014	Sweden	Cohort	43 (42)	8	Nutrition	52	CIMT	L
Montero [55]	2014	France	Cohort	38 (60)	14	Nutrition	16	FMD	L
Abu-Kishk [72]	2014	Israel	Cohort	36 (38)	15.1	Combined	24	FMD	M
Starkoff [24]	2013	USA	Cohort	34 (44)	15	Physical activity	6	Pleth	M
Hiisijärvi [47]	2019	Finland	Cohort	31 (52)	17.5	Physical activity	4	PWV	M
Hansen [23]	2013	Portugal	Cohort	31 (23)	10	Physical activity	12	RHI, PWV	M
Thopy [57]	2011	USA	Cohort	27 (74)	13	Nutrition	24	FMD	L
Nourse [26]	2015	USA	Cohort	20 (55)	14.5	Physical activity	12	RHI, PWV	M
Bond [28]	2015	UK	Cohort	20 (50)	14.1	Physical activity	2	FMD	M
Bond [29]	2015	UK	Cohort	16 (44)	14.3	Physical activity	2	FMD	M
Kallerman [8]	2019	Sweden	Cohort	15 (46)	15	Physical activity	52	PWV	M

*AIMT* aortic intima media thickness, *FMD* flow mediated dilatation, *IMT* intima media thickness, *L* low, *M* medium, *N* no, *pleth* plethysmography, *PWV* pulse wave velocity, *RCT* randomised controlled trial, *RHI* reactive hyperaemia index

Of the 72 studies, 34 (46%) studies reported endothelial function (27 (79%) flow mediated dilatation (FMD); 5 (7%) reactive hyperaemia index; 2 (6%) plethysmography). For assessment of vascular structure, 29 (39%) examined IMT (27 (93%) CIMT and 1 (3%) aortic IMT). For assessment of arterial stiffness, 30 (41%) measured pulse wave velocity (PWV). Of these, only 7 (23%) measured carotid-femoral PWV. The median (range) number of methods of measuring vascular structure and function per study was 1 [1, 3]. Studies were more likely to identify a significant improvement in vascular structure and function when measuring endothelial function (*n* = 28, 82% of total) compared to IMT (*n* = 16, 53% of total) or arterial stiffness (*n* = 19, 63% of total) (*p* = 0.03).

The interventions examined were physical activity alone in 40 (55%); dietary intervention alone in 12 (16%); dietary supplements in 3 (4%); a combination of lifestyle measures in 9 (12.5%); and various methods of drug therapy in 9 (12.5%). The median duration of intervention was 0.5 years (0, 15). In general, there was no association between duration of intervention and likelihood of finding an improvement in vascular function (r^2^ = 0.01, *p* = 0.2).

### Physical activity interventions

Physical activity interventions improved markers of vascular health in 29 out of 40 (74%) included studies (Table 1) and of these 29, 16 (55%) were school-based interventions. Although the interventions varied, 12 of the 29 studies (41%) included a structured exercise programme performed 3 times per week [8–18]. Fifteen of these 29 studies recruited only obese or overweight children and found improvements in terms of IMT and FMD [14–22] were consistent, but not in arterial stiffness or other markers of endothelial function [23–27]. In non-obese children, all 7 studies of exercise intervention, ranging from resistance training to active dance-based video game use, which measured FMD demonstrated significant increases in endothelial function [28–33]. Of the 9 studies [34–42] which measured CIMT, 2 (22%) found a decrease in IMT with exercise training, one with shuttle run training [34] and one finding that an increase in intensity and duration of any form of exercise resulted in improvements [35]. Results were inconsistent in the 12 studies, which measured PWV, with 6 (50%) [10, 12, 35, 43–45] finding reductions in arterial stiffness and 6 (50%) [39–42, 46, 47] finding no differences after the introduction of the exercise intervention. One study examined central retinal arteriolar and venular vessel diameters as early markers of vascular dysfunction and found that these reduced after an 18 month school-based physical activity programme [48].

### Dietary interventions

Of the 12 studies (Table 1) which introduced dietary interventions, nutritional alterations included increasing fatty acids [49]; increasing casein [50]; increasing ketones [51]; and increasing protein intake [52, 53] with no significant differences found. The introduction of low calorie diets reduced IMT and arterial stiffness [54], but did not affect endothelial function [55]. The Dietary Interventions to Stop Hypertension (DASH) programme, consisting of face-to-face dietetic input and a specialised meal plan was found to improve endothelial function in adolescents [56, 57]. In comparison, the Special Turku Coronary Risk Factor Intervention Project for Children (STRIP) studies, incorporating 1042 participants found that counselling alone from dietetic professionals regarding low cholesterol/low saturated fat diets introduced in infancy and maintained at 3–12 monthly intervals until 20 years of age significantly increased FMD and decreased IMT [6, 58, 59], without specific meal plans. That said, the increase in FMD was only established in boys (*p* = 0.0034) and not in girls (*p* = 0.69) [59].

### Drugs and dietary supplements

Single studies assessing the impact of alpha-lipoic acid [60], extract of citrus fruit peels [61], exenatide [62], folic acid [63] and vitamin C [64] found no statistically significant changes in vascular function (Table 2). One study of 67 obese adolescents (30% female) aged between 11–18 years reported that a 10 week prescription of orlistat 120 mg three times daily improved endothelial function, as measured by FMD, as well as reducing bodyweight, BMI and fasting total and LDL-cholesterol when combined with dietary control alone (% change in FMD 1.0 (0.5–1.6) vs 0.1 (−0.1–0.4, *p* < 0.001) [65]. Two studies examined the effects of vitamin D supplementation 2000 IU per day orally [66] and 120,000 IU per month via intramuscular (IM) injections [67]. There were no statistically significant differences reported in the IM group [67] but arterial stiffness was reduced in the high dose oral supplementation group (5.41 ± 0.73 m/sec at baseline vs 5.33 ± 0.79 m/sec after 16 weeks, *p* = 0.031) [66]. Two studies examined the effects of metformin; 1 measuring CIMT (850 mg once daily for 24 months) [68] and 1 performing echocardiography to measure arterial elasticity and systemic vascular resistance (500 mg twice daily for 22 months) [69]. Both recruited obese young people and found that metformin increased weight loss and resulted in improvements in their chosen measure of vascular function, however, both also included dietary and physical activity interventions as part of the programme.

**Table 2:** Strength of evidence of intervention to improve vascular function.

Intervention	References	Total no of participants	% of female participants	No (%) of studies reporting sex ratio	No (%) of RCTs	Change in IMT	Change in endothelial function	Change in arterial stiffness
Physical activity	[9–14,16–48,61, 90]	7588	1731	8 (20)	21 (52)	↓	↑	+/−
Protein	[51, 52]	3504	43	0	2 (100)	No	–	–
Dietary counselling regarding low fat diet	[6, 58, 92]	1062	48	1 (33)	0	↓	↑	–
Combined diet and exercise	[16,69–76]	668	39	0	3 (33)	↓	↑	↓
Fatty acids	[48]	616	49	0	0	–	–	No
Vitamin D	[65, 66]	238	37	0	1 (100)	–	–	+/−
Casein	[49]	203	62	0	0	–	–	No
DASH diet	[55, 56]	186	35	0	1 (50)	–	↑	–
Orlistat	[64]	108	29	0	1 (100)	–	↑	–
Citrus fruits	[60]	90	39	0	1 (100)	–	No	–
Alpha lipoic acid	[59]	88	28	0	1 (100)	No	No	No
Metformin	[67, 68]	74	51	0	2 (100)	↓	–	↓
Low calorie diet	[53, 54]	64	58	0	1 (50)	↓	No	↓
Folic acid	[62]	53	51	0	1 (100)	–	No	–
Ketogenic diet	[50]	43	18	0	0	No	–	–
Vitamin C	[63]	40	45	0	1 (100)	No	No	–
Exanetide	[61]	11	81	0	1 (100)	–	No	–

*DASH* Dietary Interventions to Stop Hypertension, *IMT* intima media thickness, – not studied, *RCT* randomised controlled trial.

### Combined lifestyle modification

Of the 10 studies assessing a combined lifestyle approach to improve vascular function, interventions ranged from 6 weeks to 1 year of length in children between the ages of 6–18 years (Table 2). Two studies reported no significant differences when combining dietary advice with exercise interventions [70]. One examined CIMT after 6 weeks of exercise and dietary advice and the other [71] examined FMD and PWV after 6 months of intervention [72]. Seven studies found that various combinations of diet and exercise advice reduced CIMT [73, 74] and arterial stiffness [75, 76] and increased endothelial function [16, 73, 77, 78]. None of these studies reported sex differences.

### Evidence of sexual dimorphism

Of the included studies, 6292 (40%) of the participants were female with 70 studies (95%) including both boys and girls in their study design. However, only 6 (8%) of these studies provided data for girls and boys separately, enabling assessment of sexual dimorphism in response to the intervention [28, 30, 33, 35, 38, 59]. In terms of sex differences, baseline artery diameter was reported to be different between boys and girls [33]. and an increase in FMD after moderate to vigorous physical activity was only detected when the absolute FMD value was adjusted for baseline diameter at rest. One study of 427 children and adolescents (14.0 ± 1.4 years) found that physical activity resulted in different effects in boys and girls, with increased training duration associated with increased IMT with increased arterial compliance and reduced arterial stiffness in boys, whereas only arterial compliance was affected in girls [35]. Of note, baseline arterial diameter was higher in boys compared to girls [35]. Bond et al. reported that high intensity exercise increased FMD and reduced oxidative stress, as well as providing high rates of enjoyment of the intervention in both girls and boys. Girls also demonstrated reductions in postprandial lipaemia, although this was not seen in boys [28]. Endothelial function, as measured by reactive hyperaemia index was also reported to be increased in girls compared to boys, with further increases observed in those who undertook more physical activity [30]. IMT was reported to be increased in overweight and obese patients at baseline in a group of 212 adolescents in Bohm et al. [38], but not when adjusted for sex and age. The introduction of a 4-week hospital based intervention did not improve IMT in boys or girls [37].

## Discussion

Interest in interventions to improve vascular structure and function in children and young people is growing, as evidenced by the number of studies investigating interventions to improve this in children. Given it is well established that early modification of cardiovascular risk factors improves cardiovascular outcomes later in life [8, 79], there is a public health need to study these further.

Meta-analysis was not possible within this systematic review due to differences in study methodology and methods of assessing vascular function. For example, there are many different ways to measure PWV [80], although evidence suggests different devices are likely to produce similar results [81]. To date carotid-femoral PWV has been shown to link to risk factors for CVD [82]. However, under a quarter of studies reporting arterial stiffness measured carotid-femoral PWV, with others using alternative methods such as carotid-radial PWV. As such, even available PWV data are difficult to compare.

In addition, there was a preponderance of studies focussing on obesity. This review did not consider interventions on vascular function in particular chronic health conditions, as it is likely that due to differing underlying mechanisms of disease, that the success of interventions may not represent the likelihood of success in healthy children. Studies assessing the effects on obese children were included, due to the high and rising prevalence of children being overweight internationally [83]. For any population-based initiative to be effective, it will have to improve vascular function in children with obesity, as well as normal weight children.

Our review suggests, however, that exercise may be a consistent method of improving endothelial function in children and adolescents. This is in agreement with other recent systematic reviews demonstrating that physical activity interventions produce improvements in body weight and physical fitness in preschool age children [84] and that high intensity exercise at any stage in the lifespan can improve cardiometabolic parameters [85]. Whilst the studies generally reported good adherence with the exercise interventions, most of the interventions were of <6 months duration. It is not clear whether adherence would continue with longer-term interventions. The techniques used in the included studies remain as research tools, rather than part of standard clinical practice. As such, it is not clear whether any improvements in vascular function are clinically translatable. The studies did not report having run in/washout periods and it is currently also not known how long it is likely that any changes in IMT, FMD, or PWV would persist after the interventions. This would be an increasing focus for future work. Further studies should, therefore, investigate the optimal mode, duration and intensity of exercise, with the realisation that this may vary at different stages of childhood. Based on the limited data available, however, we would suggest initiation of school based physical activity interventions of medium-high intensity at least three times per week for 60 min for optimal vascular function in both boys and girls throughout childhood and adolescence. Physical activity is likely to increase endothelial function, reduce arterial stiffness, increase arterial compliance and slow the progression of lipid accumulation in the carotid and aortic intima, delaying vascular ageing and mitigating cardiovascular risk [86], although it is not clear whether the underlying mechanisms for these changes may differ between boys and girls.

In our review, studies investigating endothelial function were more likely to detect a difference between groups. The effects of intervention were measured at variable time points, ranging from immediately afterwards to 15 years later. FMD has been shown to reduce within 30 min of exercise, whereas it remains unclear how long it might take for changes in arterial stiffness or vascular remodelling to be evident and as such, study design is important when comparing the effects of these interventions [87].

The fact that <10% of studies in children and young people report data from boys and girls separately is perhaps surprising. Reference data for blood pressure, PWV and CIMT are different for boys and girls [88–90]. It is also been reported that girls have higher FMD than boys [91]. Boys are reported to have higher baseline arterial diameters compared to girls and therefore reduced capacity to dilate [92, 93]. The effects of oestrogens and androgens on the vasculature have been studied widely, with the consensus that both can affect the vasculature and regulation of blood pressure significantly [3, 94]. It is therefore likely that these will influence vascular function as children progress through puberty into adulthood. It is recommended that sex-specific data are represented for all health conditions [95], as it was realised that historically women have been excluded from major clinical trials [96]. As a result, the sexes should be considered separately in any study, but particularly those including children from the age of adolescence onwards.

In conclusion, school-based physical activity interventions are most likely to result in improvements in endothelial function. Endothelial function may be the first variable of vascular function to change secondary to an intervention and therefore should be considered in studies looking to assess the vasculature in children. The number of studies which considered the effects of interventions on vascular function in girls and boys separately is low. Standardisation of reporting of differences between the sexes is essential to be able to ensure interventions are equally effective for boys and girls.

## Summary

### What is known about this topic

- Vascular function is different in boys and girls.
- There are several different methods of assessing vascular structure and function in children including pulse wave velocity, carotid intima media thickness and flow mediated dilatation.
- It is not clear how to improve cardiovascular function in children.

### What this study adds

- School-based physical activity interventions are most likely to result in improvements in vascular function.
- Endothelial function may be the first variable of vascular function to change secondary to an intervention.
- Standardisation of reporting of differences between the sexes is essential to be able to ensure interventions are equally effective for boys and girls.

## Data Availability

Data is available upon reasonable request.
